# The associations between pretreatment neutrophil-to-lymphocyte ratio, sarcopenia and frailty in older patients with head and neck cancer

**DOI:** 10.1016/j.jarlif.2025.100022

**Published:** 2025-08-05

**Authors:** C.D.A. Meerkerk, M.H. Emmelot-Vonk, S. Haitjema, R. de Bree

**Affiliations:** a Department of Head and Neck Surgical Oncology, University Medical Center Utrecht, Utrecht University, , House Postal Number Q.05.4.300, PO BOX 85500, 3508, GA, Utrecht, the Netherlands; b Department of Geriatric Medicine, University Medical Center Utrecht, Utrecht University, Utrecht, the Netherlands; c Central Diagnostic Laboratory, University Medical Center Utrecht, Utrecht University, Utrecht, the Netherlands

**Keywords:** Neutrophil-to-lymphocyte ratio (NLR), Comprehensive geriatric assessment, Frailty, Sarcopenia

## Abstract

**Background:**

Performing a Geriatric Assessment (GA) is recommended to help guiding treatment decisions in vulnerable older patients. As a full GA is time consuming and expensive, frailty screening methods are worth investigating. Sarcopenia and the neutrophil-to-lymphocyte ratio (NLR) could be easily available biomarkers related to frailty.

**Objectives:**

We investigated the relationships between NLR, sarcopenia, and frailty, and assessed the potential of NLR as a frailty screening method in older patients with head and neck cancer (HNC). In addition, the relationship between the NLR and each GA item was investigated.

**Design:**

Retrospective study

**Participants and Measurements:**

148 older (≥ 60 years) patients with HNC who had undergone pretreatment a GA, routine blood sample, handgrip strength (HGS) and head and neck CT or MRI for skeletal muscle mass measurement (SMM). The GA to determine frailty was assessed as outcome. Sarcopenia was defined as the combination of low SMM and low HGS.

**Results:**

The mean age was 70 (6.08 SD) years. A total of 95 (64 %) patients had an elevated NLR and 21 (14 %) had sarcopenia. Based on the GA, 56 (38 %) patients were determined as frail. Patients with an elevated NLR were more likely to be frail and sarcopenic compared to patients with a normal NLR. NLR score showed a significant though weak correlation with frailty (*r*= - 0.287, *p* < 0.05). Using GA as reference standard, the sensitivity, specificity, positive predictive value, negative predictive value and accuracy to predict frailty for NLR were 83 %, 47 %, 49 %, 83 % and 61 %, respectively. In multivariate regression analysis, the significant predictors for frailty were comorbidity, skeletal muscle index (SMI), and NLR. For elevated NLR, SMI and frailty were predictors. Elevated NLR was associated with nutritional status (OR 3.56, *P* = 0.02) and comorbidity (OR 3.81, *P* = 0.02) as independent GA items.

**Conclusion:**

Increased NLR is frequently observed in older HNC patients, often in combination with low SMI and frailty. There is a significant correlation between NLR and frailty. However, the accuracy of NLR to predict frailty based on GA is limited.

## 1 Introduction

Head and neck cancer (HNC) is increasingly diagnosed in older individuals, and as the global population rises, the incidence of HNC is projected to rise with 60 % in 2030 [1]. Evidence-based treatments for HNC involve surgery, radiotherapy (RT), chemotherapy, or a combination of these approaches. Older patients often exhibit reduced tolerance and experience more severe therapy-related side effects compared to younger patients [2]. Identification of frail older patients is essential, because it guides the customization of their treatment plans and serves as a predictor of perioperative mortality and morbidity [[3], [4], [5]]. The most appropriate method for detecting frailty is a geriatric assessment (GA). It evaluates a patient’s functional, nutritional, cognitive, mood, physical, and comorbidity status [6].

Sarcopenia, defined by the European Working Group on Sarcopenia in Older People (EWGSOP), involves both low muscle function (e.g., handgrip strength, HGS) and low muscle quantity (e.g., skeletal muscle mass, SMM) [7]. Sarcopenia is predictive and prognostic for adverse health outcome and overall survival in HNC patients [8]. Recent research indicates that frail patients are more likely to be sarcopenic [9]. However, there is growing consensus that although sarcopenia may be a component of frailty, frailty is a broader concept than sarcopenia alone. Therefore sarcopenia could not be used to detect frailty, or be used interchangeable for other frailty screening methods like the Geriatrics 8 (G8) [10,11]. So, exploring other biomarkers may enhance patient selection for a full frailty assessment by GA. Selection of patients for a GA may be particularly important, because GA is very laborious and resources are scarce.

One of the simplest and most readily available clinical blood tests is the complete blood cell count, which reports among other things the absolute neutrophil count (ANC) and absolute lymphocyte count (ALC). The neutrophil-to-lymphocyte ratio (NLR), calculated by dividing ANC by ALC, serves as an index for assessing the systemic inflammatory response in critically ill patients [[12], [13], [14]]. NLR may reflect systemic inflammation in cancer patients and their immune system’s ability to combat malignant cells. Recent reports suggest that NLR can serve as a prognostic marker for adverse health outcomes in various malignancies [15,16]. While NLR has been rarely examined in older HNC patients, recent findings indicate that it is also an independent prognostic marker in these patients [17].

This study aims to explore the relationships between NLR, sarcopenia, and frailty, and assess the potential of NLR for frailty screening in older patients with HNC. We also investigated the relationship between the NLR and each GA item separately.

## 2 Materials and methods

### 2.1 Ethical approval

The design of this study was approved by the Medical Ethical Research Committee of the University Medical Center Utrecht (approval ID 17–365/C). Informed consent was waived because of its retrospective character. All procedures in this study were in accordance with the ethical standards of the institutional and/or national research committee and with the 1964 Helsinki declaration (Version 2008) and its later amendments or comparable ethical standards. All data were handled according to general data protection regulation (GDPR).

### 2.2 Patients and study design

In this single-center retrospective study, older patients (≥ 60-years old) with pathologically proven HNC treated in the University Medical Center Utrecht, Utrecht, The Netherlands, between September 2018 and January 2020 were reviewed. Patients with a GA, HGS measurement, pre-treatment CT or MRI during their diagnostic workup, and a routine pre-operative blood sample were included in this study. Older patients with HNC were offered geriatric assessment, but patients could refuse. As a consequence, not all older patients underwent frailty assessment and could be included. Patients with histologic tumor types other than squamous cell carcinoma were excluded.

**Fig. 1:**
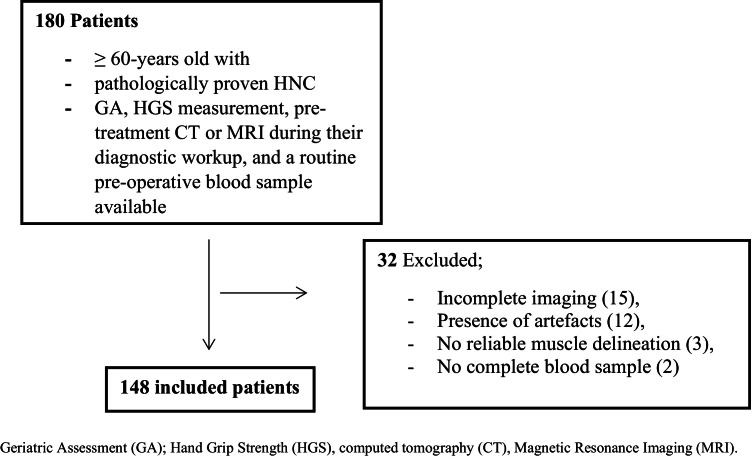
Flowchart of the patient selection.

This resulted in an initial inclusion of 180 patients. Patients were excluded due to insufficient quality of diagnostic imaging for SMM assessment (incomplete imaging at time of diagnoses (fifteen), presence of artefacts on CT or MRI (twelve), no reliable delineation between muscle and surrounding tissue which impaired measurements of skeletal muscle mass (three)) and no complete blood sample for NLR assessment (two). This resulted in the final inclusion of 148 patients (Fig. 1).

Relevant demographic and clinical variables were collected from patients medical record: age, sex, weight loss in the past six months, body mass index (BMI), smoking status, alcohol use, comorbidity as evaluated by the Adult Comorbidity Evaluation-27 index (ACE-27), tumor localization, tumor type (primary, second primary or recurrence), histology, the TNM stage according the 8th edition of the UICC tumor classification of malignant tumors, treatment intention (curative or palliative), treatment modality and imaging technique (CT or MRI).

### 2.3 Frailty

The GA, carried out pre-operatively, included instruments to measure functional status, nutritional status, cognition, mood, physical function, and comorbidity. Functional status was assessed by a modified version of the Katz-15 Index of Independence that measures the Basic Activities of Daily Living (ADL) and the Instrumental Activities of Daily Living [18]. ADL was assessed using the six items equal to the Katz-6 index [19] (i.e., bathing, dressing, eating, toileting, continence, transferring), the nine resting items (partly adapted from the Lawton IADL index [20]) were used to assess IADL (i.e., traveling, grooming, preparing a meal, use of telephone, shopping, household tasks, managing medications, managing finances, and mobility). The Malnutrition Universal Screening Tool (MUST) was used to assess nutritional status [21]. Cognition was assessed with the mini-mental state examination (MMSE) [22]. Mood was assessed with the Patient Health Questionnaire-2 (PHQ-2) [23] followed by a Geriatric Depression Scale 15 (GDS-15) [24] in case of a positive PHQ-2. Physical function was assessed with 4-meter walk test (4MWT) [25]. The Charlson Comorbidity Index (CCI) was used to assess the comorbidity [26]. Point for age and their current malignancy were not included calculating the CCI since this involved every patient.

Each instrument was defined as deficient according to validated cutoff scores: Katz-6 ≥ 1, IADL ≥ 1, MUST ≥ 1, MMSE < 24, PHQ-2 ≥ 1, 4MWT > 1 s per meter, and CCI ≥ 2. Overall, a patient was defined frail if the GA had an deficient outcome on at least two of the seven instruments used.

### 2.4 Neutrophil-to-lymphocyte ratio

The complete blood counts were obtained from the Utrecht Patient Oriented Database, where a full complete blood count is stored for every patient that underwent routine hemocytometry in the University Medical Center Utrecht since 2003 [27]. The NLR was calculated by dividing the absolute neutrophil count (ANC) by the absolute lymphocyte count (ALC). Tumor cells have been found to secrete cytokines that stimulate the bone marrow to produce more neutrophils. In patients with head and neck cancers, an elevated neutrophil-to-lymphocyte ratio (NLR) has been identified as a negative prognostic marker for survival outcomes, as demonstrated in several meta-analyses. Based on those studies and of Tham et al. conducting especially in patients with HNC, we considered a NLR >2.5 as high NLR considering the outcome of adverse health events [15,[28], [29], [30]].

### 2.5 Sarcopenia

As recommended by the EWGSOP2-criteria we used the combination of low muscle function, as determined by handgrip strength measurements, and low muscle quantity, as determined by SMM, for the diagnosis of sarcopenia [7].

#### 2.5.1 Muscle function

Overall muscle function is strongly related to handgrip strength (HGS) [31]. HGS was measured using a Jamar hydraulic handheld dynamometer according to the recommendations of the American society of hand therapists (ASHT) and expressed in kilograms (kg). Patients were asked to squeeze maximally with each hand. The average score of the left and right hand tests was used for analysis. Patients had low HGS if the HGS was below twenty-seven kg (men) or below sixteen kg (women) [7].

#### 2.5.2 Skeletal muscle mass

For assessment of SMM the cross-sectional muscle area (CSMA) at the level of the third cervical vertebrae (C3) on CT or MRI imaging was measured in all patients before initiating treatment. The axial slice of the imaging which showed both transverse processes and the entire vertebral arc was selected for the segmentation of muscle tissue. For CT imaging, muscle area was defined as the pixel area between the radiodensity range of – 29 and + 150 Hounsfield units (HU), which is specific for muscle tissue [32]. For MRI, muscle area was manually segmented, and fatty tissue was manually excluded. Segmentation of muscle tissue was manually performed using the commercially available software package SliceOmatic (version 5.0, Tomovision, Canada). The first author (CM) performed SMM measurements in all 148 patients.

The cross-sectional muscle area at the level of C3 was converted to CSMA at the level of L3 using a formula published by Swartz et al. [33]. The lumbar skeletal muscle index (SMI) was calculated by correcting skeletal muscle mass at the level of L3 for height. Patients had a low SMI if this value was below 43.2 cm^2^/m^2^; this cut-off value was established in a separate cohort of patients with HNC [34].

### 2.6 Statistical analysis

Data analyses were performed using IBM SPSS statistics 26. Baseline clinical characteristics were collected and continuous data are represented as mean ± standard deviation (SD). Categorical data are represented as a number and percentage from total. Frailty was based on the GA and is presented dichotomously as frail and non-frail based on previously published cut-offs for the GA. NLR was presented dichotomously as normal and elevated based on the previously published cut-off value of 2.5 [28]. The SMM, was presented dichotomously as low SMI and normal SMI based on a previously published specific cut-off value for SMI of 43.2 cm^2^/m^2^ [34]. Muscle function was presented dichotomously as low muscle function and normal muscle function based on previously published gender-specific cut-offs for HGS [7]. Sarcopenia was presented dichotomously as sarcopenic (if patients had both a low muscle function and low SMI) and non-sarcopenic (all other patients).

Independent sample *t*-tests or Chi-square statistics were used for analyzing differences between the frequencies of each categorical variable with the presence or absence of elevated NLR. For calculating the correlation between NLR, SMI, HGS and frailty score (number of deviating items), continuous scores were used to analyze with the bivariate Pearson’s r-correlation coefficients.

Univariate logistic regression analyses were performed, with frailty according to GA or NLR as dependent variables and the baseline variables as independent variables. Variables were selected based on clinical relevance. Variables that were statistically significant (*p* < 0.05) in the univariate regression were included in the multivariate logistic regression with odds ratios (ORs) and 95 % CI’s provided. We evaluated collinearity among the variables included in the models by calculating the Variation Inflation Factor (VIF). There is no universally accepted threshold for VIF to indicate multicollinearity, as the empirical literature reports a range of values. For instance, Vittinghoff et al. proposed a threshold of 10, while Johnston et al. adopted a more conservative cutoff of 2.5 [35]. Given that multicollinearity poses a greater concern in studies with smaller sample sizes, we arbitrarily adopted a threshold of 3.5, which is commonly used to indicate a substantial risk of multicollinearity [36].

The sensitivity, specificity, negative predictive value (NPV), and positive predictive value (PPV) of NLR and, sarcopenia to predict frailty according to the GA were calculated from a 2 × 2 cross-table. Confidence intervals (95 % CI) are reported.

## 3 Results

### 3.1 Patient characteristics

This study consisted of 148 patients with HNC diagnosed between September 2018 and January 2020 and included 100 men (68 %) and 48 women (32 %) with a mean age of 70 (SD 6.08) years. Patient characteristics are presented in Table 1. Stage IV was the most common TNM stage (43 %). Most of the patients (36 %) had mild comorbidities according to the ACE-27 score. Of the included patients, the GA defined 56 (38 %) patients as frail. The majority of the patients (61 %) had low SMI at diagnosis. Low HGS at diagnosis was seen in a minority of the included patients (22 %). Of the included patients, 21 patients (14 %) were sarcopenic (i.e., combination of low SMI and low HGS). The majority (64 %) had an elevated NLR at diagnosis. The mean time interval in which the NLR blood sample, GA and HGS measurement (first consultation) and CT/MRI scan were performed was 1.8 (SD 0.8) weeks. The appendix shows boxplots with the distributions of NLR, LSMI, HGS, and G8.

**Table 1:** Characteristics of older HNC patients with and without elevated NLR (>2.5).

	Total		NLR >2.5		NLR normal		*χ*^2^	*p-value*
	*N* = 148		*N* = 95		*N* = 53			
Age (years) (M, SD)	70.36	6.08	70.81	6.93	69.57	6.54	N.A.	N.A.
								
Sex (n, %)								
*Male*	100	68	63	66	37	70	0.19	0.66
*Female*	48	32	32	34	16	30		
Weight loss 6 months prior to diagnosis (n, %)								
*Non*	116	78	73	77	43	81	1.49	0.47
*<10 %*	25	17	16	17	9	17		
*≥ 10 %*	7	5	6	6	1	2		
BMI (kg/m^2^) (n, %)								
*<20*	9	6	6	6	3	6	3.46	0.33
*20–24.9*	53	36	39	41	14	26		
*25–29.9*	63	43	37	39	26	49		
*≥ 30*	23	16	13	14	10	19		
Smoker (n, %)								
*No*	26	18	18	19	8	15	1.31	0.52
*Former*	72	49	48	51	24	45		
*Current*	50	34	29	31	21	40		
Alcohol use (n, %)								
*No*	29	20	21	22	8	15	2.78	0.25
*Yes*	99	67	59	62	40	75		
*Former*	20	14	15	16	5	9		
ACE-27 score (n, %)								
*Non*	48	32	27	28	21	40	10.61	0.01
*Mild*	53	36	29	31	24	45		
*Moderate*	29	20	24	25	5	9		
*Severe*	18	12	15	16	3	6		
Localization (n, %)								
*Oral cavity*	31	21	22	23	9	17	2.76	0.95
*Nasal cavity*	6	4	3	3	3	6		
*Nasopharynx*	5	3	3	3	2	4		
*Oropharynx*	37	25	25	26	12	23		
*Hypopharynx*	11	7	6	6	5	9		
*Larynx*	29	20	17	18	12	23		
*Salivary glands*	17	11	12	13	5	9		
*Skin*	2	1	1	1	1	2		
*Unknown primary*	10	7	6	6	4	8		
Type of tumor (n, %)								
*Primary*	141	95	90	95	51	96	0.58	0.75
*Recurrent*	1	1	1	1	0	0		
*Second primary*	6	4	4	4	2	4		
Histology (n, %)								
*Squamous cell carcinoma*	117	79	75	79	45	85	1.50	0.47
*Adenocarcinoma*	18	12	10	11	8	15		
*Other*	13	9	10	11	3	6		
TNM Stage (n, %)								
*I*	23	16	18	19	5	9	5.44	0.27
*II*	29	20	15	16	14	26		
*III*	33	22	21	22	12	23		
*IV*	63	43	42	44	21	40		
Type of imaging (n, %)								
*CT*	91	61	59	62	32	60	0.04	0.84
*MRI*	57	39	36	38	21	40		
*Treatment intention (n, %)*								
*Curative*	138	93	85	89	53	100	5.98	0.01
*Palliative*	10	7	10	11	0	0		
*Treatment type (n, %)*								
*CRT*	24	16	13	14	11	21	8.89	0.18
*Radiotherapy*	63	43	36	38	27	51		
*Surgery only*	26	18	21	22	5	9		
*Surgery w adjuvant radiotherapy*	28	19	19	20	9	17		
*Surgery w adjuvant CRT*	3	2	3	3	0	0		
*No treatment*	4	3	3	3	1	2		
*Frail (n, %) according to GA*								
*No*	92	62	48	51	44	83	15.270	< 0.001
*Yes*	56	38	47	49	9	17		
*Low muscle function (n, %)*								
*No*	115	78	71	75	44	83	1.347	0.25
*Yes*	33	22	24	25	9	17		
*Low SMI (n, %)*								
*No*	57	39	29	31	28	53	7.147	0.003
*Yes*	91	61	66	69	25	47		
*Sarcopenia (n, %)*								
*No*	127	86	77	81	50	94	4.933	0.03
*Yes*	21	14	18	19	3	6		

BMI body mass index, ACE-27 adult comorbidity evaluation-27, TNM tumor node metastasis., CT computer tomogram, MRI magnetic resonance imaging, CRT chemoradiotherapy, SMI skeletal muscle index, NLR neutrophil lymphocyte ratio.

Table 1 shows an overview of differences between patients with and without an elevated NLR (>2.5). Patients with an elevated NLR were more likely to be frail according to the GA (49 % versus 17 %; *p* < 0.001), to have low SMI at diagnosis (69 % versus 47 %; *p* < 0.01), to be sarcopenic (combination of low HGS and low SMI) at diagnosis (19 % versus 6 %; *p* < 0.05), to have a moderate or severe comorbidity defined by the ACE-27 score (25 % versus 9 %; *p* < 0.05), to have a palliative treatment intention (11 % versus 0 %; *p* < 0.05).

### 3.2 Correlation analysis of NLR, HGS, SMI and frailty

NLR showed a significant though weak correlation with the number of deviating GA items (*r*= - 0.287, *p* < 0.05). NLR showed a significant though weak correlation with HGS (*r*= - 0.176, *p* < 0.05) and a significant though weak correlation with SMI (*r*= - 0.220, *p* < 0.05).

SMI and HGS showed significant though weak correlations with the number of deviating GA items (*r* = 0.241, *p* < 0.01 and *r* = 0.263, *p* < 0.01, respectively).

### 3.3 Univariate and multivariate logistic regression

Univariate and multivariate logistic regression analyses with frailty according to the GA and elevated NLR as the dependent variables were performed.

Table 2 shows the univariate regression analysis with frailty according to GA as the dependent variable which distinguished ACE-27 score (OR 9.17, 95 % CI 4.58–28.60, *P* < 0.001), low HGS (OR 0.93, 95 % CI 0.90–0.97, *P* < 0.001), low SMI (OR 0.92, 95 % CI 0.87–0.96, *P* < 0.001), sarcopenia (OR 2.86, 95 % CI 1.10–7.42, *P* = 0.03), and elevated NLR (OR 4.58, 95 % CI 2.06–10.19, *P* < 0.001) as significant variables for predicting frailty by GA. These significant variables were subjected to two different multivariate analyses to predict GA. The first with sarcopenia and the second with hand grip strength and SMI separately because of assumed multicollinearity. This was based on calculating the VIF among the variables included in the models and it distinguishes sarcopenia, hand grip strength and SMI for collinearity (VIF ≥ 3.5). In the first multivariate analysis ACE-27 score (OR for severe ACE 7.47, 95 % CI 1.67–23.38, *P* < 0.001) and elevated NLR (OR 3.64, 95 % CI 1.53–8.65, *P* = 0.003) remained significant. In the second ACE-27 score (OR 14.72 95 % CI 4.62–29.60, *P* < 0.001), low SMI (OR 0.91, 95 % CI 0.85–0.97, *P* = 0.004) and elevated NLR (OR 3.08, 95 % CI 1.23–7.71, *P* = 0.02) remained significant.

**Table 2:** Univariate and multivariate logistic regression analysis for analyzing variables associated with frailty (based on the GA) in older patients with HNC.

Frailty	Univariate analysis			Multivariate analysis					
	OR	95 % CI	P-value	OR	95 % CI	P-value	OR	95 % CI	P-value
Age (years)									
<70	Ref.								
*≥ 70*	1.21	0.62–2.34	0.57						
Sex									
*Male*	Ref.								
*Female*	1.85	0.92–3.74	0.12						
ACE-27 score									
*Non*	Ref.			Ref.			Ref.		
*Mild*	1.96	0.79–4.80	0.12	1.99	0.76–5.20	0.29	2.15	0.75–4.81	0.14
*Moderate*	4.67	1.34–9.21	0.003	3.53	1.21–10.24	0.004	4.91	1.21–10.60	0.007
*Severe*	9.17	4.58–28.60	<0.001	7.47	1.67–23.38	<0.001	14.72	4.62–29.60	<0.001
TNM Stage									
*I*	Ref.								
*II*	0.47	0.13–1.76	0.27						
*III*	1.68	0.54–5.18	0.36						
*IV*	2.51	0.91–6.94	0.08						
Handgrip strength	0.93	0.90–0.97	<0.001				0.98	0.94–1.03	0.396
									
SMI	0.92	0.87–0.96	<0.001				0.91	0.85–0.97	0.004
Sarcopenia									
*No*	Ref.			Ref.					
*Yes*	2.86	1.10–7.42	0.030	1.29	0.43–3.89	0.65			
NLR >2.5									
*No*	Ref.			Ref.					
*Yes*	4.58	2.06–10.19	<0.001	3.64	1.53–8.65	0.003	3.08	1.23–7.71	0.02

SMI skeletal muscle index, TNM tumor node metastasis, NLR neutrophil lymphocyte ratio, ACE-27 adult comorbidity evaluation-27.

Table 3 shows that the univariate regression analysis for elevated NLR as dependent variable distinguished ACE-27 score (OR 3.89, 95 % CI 1.1–15.22, *P* = 0.05), low SMI (OR 2.89, 95 % CI 1.44–5.81, *P* = 0.003), sarcopenia (OR 3.89, 95 % CI 1.10–13.92, *P* = 0.04), and frailty by GA (OR 4.58, 95 % CI 2.06–10.19, *P* < 0.001) as significant variables associated with elevated NLR. These significant variables were subjected to two different multivariate analyses. The first with sarcopenia and the second with SMI because of assumed multicollinearity. This was based on calculating the VIF among the variables included in the models and it distinguishes sarcopenia, hand grip strength and SMI for collinearity (VIF ≥ 3.5). In the first multivariate analysis only frailty by GA (OR 3.66, 95 % CI 1.50–8.72, *P* = 0.003) remained significant. In the second low SMI (OR 2.70, 95 % CI 1.22–5.97, *P* = 0.01) and frailty (OR 2.75, 95 % CI 1.11–6.76, *P* = 0.03) remained significant.

**Table 3:** Univariate and multivariate logistic regression analysis for analyzing variables associated with elevated NLR in older patients with HNC.

NLR	Univariate analysis			Multivariate analysis					
	OR	95 % CI	P-value	OR	95 % CI	P-value	OR	95 % CI	P-value
ACE-27 score									
*Non*	Ref.			Ref.			Ref.		
*Mild*	0,94	0.43–2.06	0.88	0.67	0.28–1.56	0.35	0.74	0.31–1.76	0.50
*Moderate*	3.73	1.22–11.44	0.02	2.28	0.70–7.41	0.17	3.07	0.89–10.47	0.07
*Severe*	3.89	1.1–15.22	0.05	1.36	0.29–6.23	0.70	1.99	0.41–9.69	0.40
Low HSG									
*No*	Ref.								
*Yes*	2.01	0.83–4.84	0.12						
Low SMI									
*No*	Ref.						Ref.		
*Yes*	2.89	1.44–5.81	0.003				2.70	1.22–5.97	0.01
Sarcopenia									
*No*	Ref.			Ref.					
*Yes*	3.89	1.10–13.92	0.04	3.04	1.54–8.72	0.12			
Frailty(GA)									
*No*	Ref.			Ref.			Ref.		
*Yes*	4.58	2.06–10.19	<0.001	3.66	1.50–8.72	0.003	2.75	1.11–6.76	0.03

SMI skeletal muscle index, HGS Hand grip strength, GA geriatric assessment, ACE-27 adult comorbidity evaluation-27. Correction for age in the uni- and multivariate logistic regression analysis.

### 3.4 The accuracy of the NLR and sarcopenia to predict frailty

Table 4 shows the sensitivity, specificity, PPV, NPV and accuracy of the NLR and sarcopenia for the diagnosis of frailty according to the GA. The NLR shows a sensitivity of 83 %, specificity of 47 %, PPV of 49 %, NPV of 83 % and accuracy of 61 %. Sarcopenia shows a sensitivity of 25 %, specificity of 92 %, PPV of 67 %, NPV of 67 % and accuracy of 67 % to predict frailty. The appendix shows the receiver operating characteristic (ROC) curve analyses of the NLR for frailty. The area under the curve(AUC) is 0.75, which is considered moderate.

**Table 4:** The sensitivity, specificity, PPV, NPV and accuracy of NLR and sarcopenia for frailty according to the GA.

	Sensitivity	Specificity	PPV	NPV	Accuracy
NLR	83 %	47 %	49 %	83 %	61 %
Sarcopenia	25 %	92 %	67 %	67 %	67 %

NLR neutrophil lymphocyte ratio, PPV positive predictive value, NPV negative predictive value.

### 3.5 Association between NLR and each GA item

As shown in Table 5 univariable analysis revealed several associations between the NLR and GA items among which are ADL (OR 4.06, 95 % CI, 1.94–14.47, *P* = 0.01), nutritional status (OR 5.50, 95 % CI, 3.07–15.15, *P* = 0.01), physical function (OR 4.37, 95 % CI, 2.03–11.32, *P* = 0.03) and comorbidity (OR 6.77, 95 % CI, 4.31–15.87, *P* = 0.002). However, the multivariable analysis revealed only nutritional status (OR 3.56, 95 % CI 1.04–11.50, *P* = 0.02) and co-morbidity (OR 3.81, 95 % CI, 1.74–10.98, *P* = 0.02) as independent factors associated GA items.

**Table 5:** Association between NLR and each item of the GA.

GA item	Univariate logistic regression			Multivariate logistic regression*		
	OR	95 % CI	*P*-value	OR	95 % CI	*P*-value
ADL	4.06	1.94–14.47	0.01	2.80	0.41–13.96	*0.22*
IADL	2.86	0.39–7.92	0.37	–	–	–
Nutritional status	5.50	3.07–15.15	0.01	3.56	1.04–11.50	0.02
Cognitive status	–	–	–	–	–	–
Mood	1.97	0.91–7.63	0.34	–	–	–
Physical Function	4.37	2.03–11.32	0.03	1.57	0.37–6.72	0.05
Comorbidity	6.77	4.31–15.87	0.002	3.81	1.74–10.98	0.02

Geriatric Assessment (GA); Odds Ratio (OR); 95 % Confidence Interval (CI); Activities of Daily Living (ADL); Instrumental Activities of Daily Living (IADL).

⁎ Including age and sex.

### 3.6 Overview of the coherence of frailty according to GA, NLR and sarcopenia

**Fig. 2:**
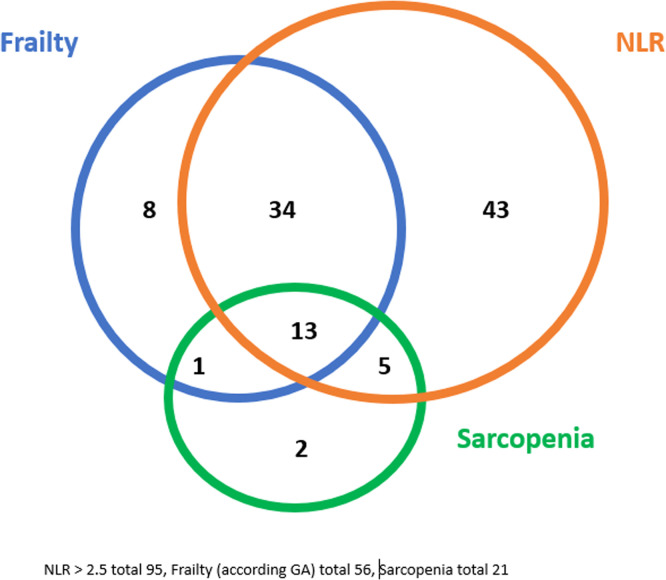
Overview of the coherence of frailty, NLR and sarcopenia.

Fig. 2 shows an overview of the coherence of frailty on GA, NLR and sarcopenia. Thirteen patients were frail, based on the GA, had a deficient NLR and were sarcopenic. Thirty-four patients were frail based on the GA, had a deficient NLR but were not sarcopenic. And 48 had a deficient NLR but were not distinguished as frail on GA.

## 4 Discussion

This retrospective study, conducted in 148 older patients with HNC, showed associations between NLR and frailty and sarcopenia. Patients with an elevated NLR were more likely to be frail and sarcopenic. NLR score showed a significant though weak correlation with frailty, SMI and HGS. In multivariate regression analysis, the only significant predictors for frailty were comorbidity, SMI, and NLR. However, the finding that comorbidity according to the ACE-27 score is predictive for frailty, may be at least partly explained by the fact that comorbidity according to the CCI was included in the GA. SMI and frailty were predictors for elevated NLR. Elevated NLR was associated with nutritional status and comorbidity as independent GA items.

In the present study NLR was used as measure for systemic inflammation. NLR showed significant though weak correlations with SMI and HGS. Local inflammation in the microenvironment of the tumor leads to chronic systemic inflammation which significantly effects the SMI. This phenomenon is known as cancer cachexia [37]. Cachexia is a complex metabolic syndrome in which systemic inflammation is the key feature and weight loss is the key diagnostic criterion. Cachexia can be an underlying condition in patients with sarcopenia. Among patients with HNC, cachexia is more pronounced as this cancer affects the functional structures of the human body that are directly involved in nutritional intake. As a result, deglutitive and masticatory functions are affected resulting in a deterioration of nutritional status [38]. NLR is not only a measure of inflammation, but also a reflection of nutritional status [39]. High NLR has proven its usefulness as a strong prognostic factor in several types of cancers [[40], [41], [42], [43]], and also as a predictor and a marker of inflammatory or infectious pathologies and postoperative complications [44,45]. This could explain why we found an association between the NLR and the malnutrition and comorbidity items of the GA.

**Fig. 3:**
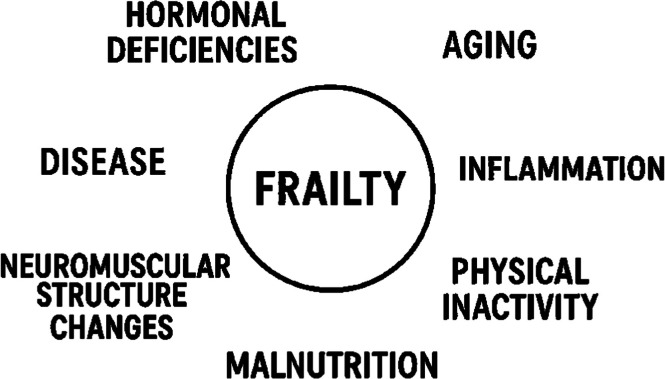
Overview of the components causing frailty.

The concept of frailty is caused by many components (Fig. 3) But the relationship between inflammation and frailty is complex since both linearly increase with advancing old age. However, there is increasing evidence that inflammation has an important role in the pathophysiology of frailty independent of age. Several studies suggested that this role is mainly based on catabolic effect of pro-inflammatory cytokines on muscles. Pro-inflammatory cytokines may influence frailty either directly by promoting protein degradation, or indirectly by affecting important metabolic pathways [46]. Oxidative stress with age is sufficient to cause DNA, lipid and muscle damage and this results in cellular and organ dysfunction [47,48]. These findings suggest that inflammation directly or indirectly contributes to the frailty pathophysiology.

In previous studies also a significant, but weak correlation between SMI and frailty was found [10,11]. As shown in Fig. 2 there is (partly) an overlay of the constructs of frailty, sarcopenia and inflammation. Elevated NLR and low SMI appeared to be independent predictors for frailty. This suggests that both entities (NLR and sarcopenia) contribute to frailty and could be underlying factors. Incorporating the NLR into pre-treatment assessments for older head and neck cancer patients holds potential clinical utility as a simple, cost-effective biomarker of systemic inflammation. Elevated NLR has been associated with poorer prognosis, reduced treatment tolerance and adverse health outcome [[40], [41], [42], [43], [44], [45]]. Integrating NLR could aid in risk stratification, inform treatment decisions, and help identify patients who may benefit from closer monitoring or supportive care interventions.

However, NLR and sarcopenia are both not suitable as screening test for GA. To select patients for GA (as opposite to exclusion), high sensitivity and negative predictive value are essential, ensuring frail patients indeed receive GA. The sensitivity and negative predictive value of NLR to be used as screening test for GA may be acceptable, but specificity and positive predictive value were too poor, meaning that too many patients are selected for GA. This means that although systematic inflammation was significantly associated with frailty and sarcopenia, the correlations are too weak to use NLR to select patients for GA.

For a GA screening test, the sensitivity and NPV are the most important characteristics: ideally all frail patients are selected for GA and the number of non-frail patients undergoing a GA is very low. The sensitivity of the NLR was modest but unfortunately the NPV was 67 %. Also, the specificity of 47 % is a limiting factor to introduce the NLR in daily practice. Besides, studies showed that the G8 shows a better sensitivity and specificity [49].

Our study has some limitations. In this study only patients who had a GA were included, which may introduce some selection and selection bias. This selection bias may have led to primarily vulnerable patients receiving a GA. As a result, it may appear that certain biomarkers are strongly associated with frailty, while this association may not hold true for fitter patients who were not assessed. The use of two different imaging techniques, CT and MRI, for the assessment of SMM may raise concerns. However, these two different imaging modalities have a significant high correlation in quantifying SMM when measured by CSA at the level of C3 [50] so we do not think this influences the found results.

Our study also has important strengths. Firstly, the study was performed in a large group of patients. Secondly, a short period between the first consultation with GA assessment, MRI or CT, and HGS measurement and quantification of SMM was achieved (1.8 weeks). Also, all the segmentation of muscle tissue was manually performed by the first author, who was blinded for outcomes of frailty, HGS and NLR. Because of an excellent inter-observer agreement for skeletal muscle mass measurement at the level of C3 was demonstrated, these SMI measurements findings can be considered to be uniformly useful [51].

In conclusion, increased NLR is frequently observed in elderly HNC patients, often in combination with low SMI and frailty. There is a significant correlation between NLR and frailty. However, the accuracy of NLR to predict frailty on GA is limited. Further research is needed to potentially improve frailty detection through a combination of biomarkers, which is also consistent with the definition of frailty as a multidimensional syndrome caused by changes in multiple biological systems. In this context, artificial intelligence (AI) could play a role. AI models with biomarkers could potentially be implemented alongside existing frailty scales in healthcare to identify frailty risks in HNC patients. This would enable early intervention in the syndrome and its associated negative health outcomes in HNC patients.

## CRediT authorship contribution statement

**C.D.A. Meerkerk:** Writing – original draft, Resources, Methodology, Investigation, Formal analysis, Data curation, Conceptualization. **M.H. Emmelot-Vonk:** Writing – review & editing, Supervision, Conceptualization. **S. Haitjema:** Writing – review & editing, Resources, Data curation. **R. de Bree:** Writing – review & editing, Supervision, Conceptualization.

## Declaration of competing interest

The authors declare that they have no conflict of interest.

## Figures and Tables

**Figure:**
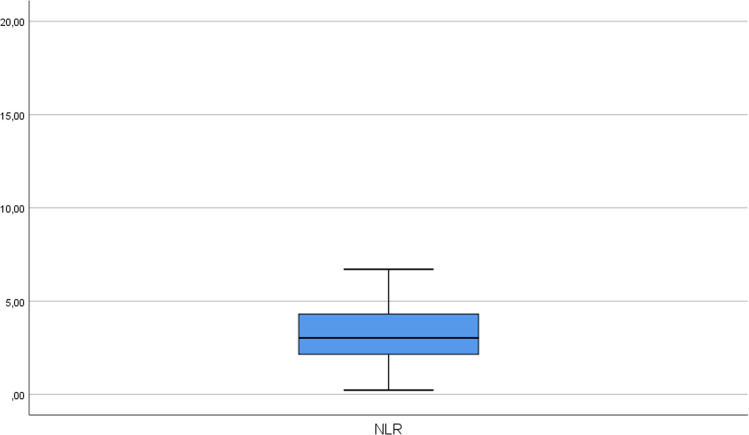


**Figure:**
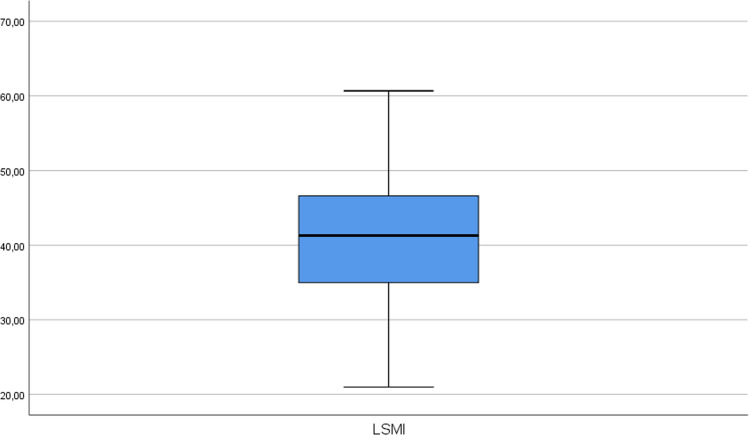


**Figure:**
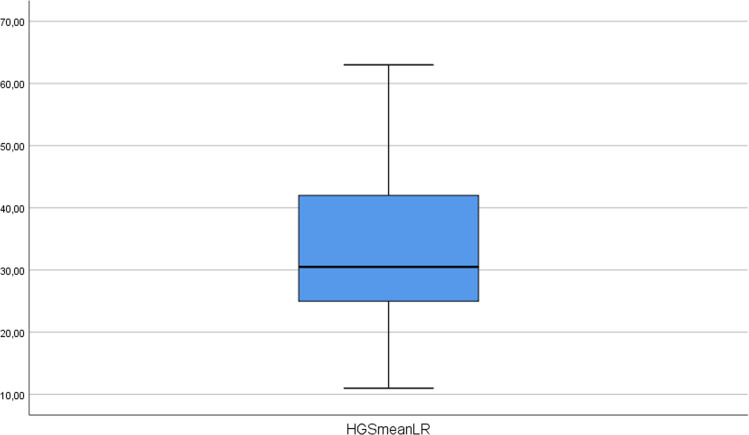


**Figure:**
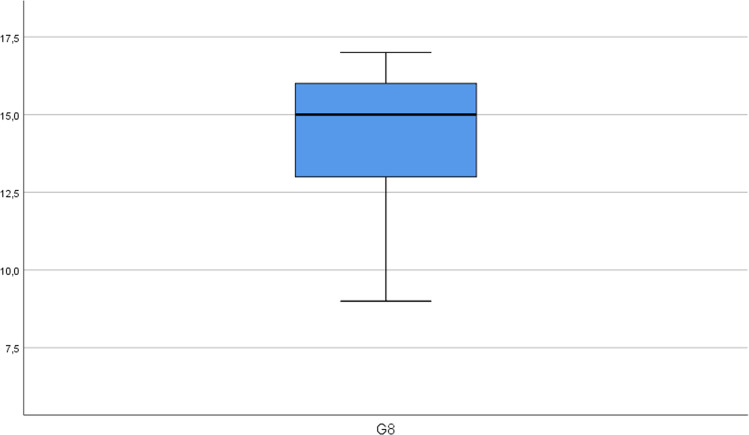


**Figure:**
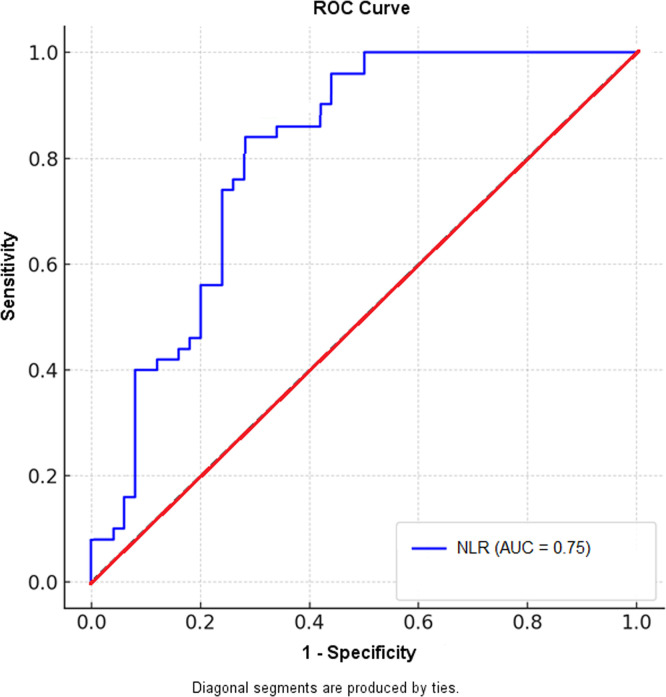


## Appendix

These boxplots show the distributions of NLR, LSMI, HGS, and G8.

*The NLR has a median of 3.03 and a mean of 3.53, with a minimum of 0.23 and a maximum of 6.97.*

*The LSMI has a median of 41.28 and a mean of 40.82, with a minimum of 20.99 and a maximum of 60.67.*

*The HSG has a median of 30.50 and a mean of 33.38, with a minimum of 11.00 and a maximum of 63.00.*

*The G8 has a median of 15.00 and a mean of 14.12, with a minimum of 9.00 and a maximum of 17.00.*

This figure shows the receiver operating characteristic (ROC) curve analyses of the NLR for frailty. The area under the curve(AUC) is 0.75.
